# Long-term survival of living donor renal transplants: A single center study

**DOI:** 10.4103/0971-4065.73439

**Published:** 2010-10

**Authors:** J. Hassanzadeh, A. A. Hashiani, A. Rajaeefard, H. Salahi, E. Khedmati, F. Kakaei, S. Nikeghbalian, A. Malek-Hossein

**Affiliations:** Department of Epidemiology, School of Health and Nutrition, Shiraz University of Medical Sciences, Shiraz, Iran; 1 Faculty of Health, Arak University of Medical Sciences, Arak, Iran; 2 Shiraz Organ Transplantation Center, Namazi Hospital, Shiraz University of Medical Sciences, Shiraz, Iran; 3 Department of Social Welfare, University of Social Welfare and Rehabilitation Sciences, Tehran, Iran; 4 Department of Surgery, Imam Reza Hospital, Tabriz University of Medical Sciences, Tabriz, Iran

**Keywords:** Cox proportional hazard model, graft survival rate, living donor, renal transplantation

## Abstract

Kidney transplantation is the treatment of choice for end-stage renal disease. The aim of this study was to determine the ten-year graft survival rate of renal transplantation in patients who have been transplanted from live donors. This is a historical cohort study designed to determine the organ survival rate after kidney transplantation from live donor during a 10-year period (from March 1999 to March 2009) on 843 patients receiving kidney transplant in the transplantation center of Namazi hospital in Shiraz, Iran. Kaplan-Meier method was used to determine the survival rate, log-rank test was used to compare survival curves, and Cox proportional hazard model was used to multivariate analysis. Mean follow-up was 53.07 ± 34.61 months. Allograft survival rates at 1, 3, 5, 7, and 10 years were 98.3, 96.4, 92.5, 90.8, and 89.2%, respectively. Using Cox proportional hazard model, the age and gender of the donors along with the creatinine level of the patients at discharge were shown to have a significant influence on survival. The 10-year graft survival rate of renal transplantation from living donor in this center is 89.2%, and graft survival rate in our cohort is satisfactory and comparable with reports from large centers in the world.

## Introduction

Chronic kidney disease (CKD) is a public health concern, imposing heavy financial burden to the society.[1] According to the data obtained from ‘Management Center for Transplantation and Special Diseases,’ number of end-stage renal disease (ESRD) patients under renal replacement therapy in Iran (population = 70 million) was about 25 000 in 2006. Considering an annual growth rate of 12%, that number can reach 40 000 year 2011. The prevalence and incidence rate of ESRD in Iran have reported to be 357 and 57 cases per million populations per year, respectively.[2] During recent years, need for renal replacement therapy has been increased in the entire world including Iran.[2] Most of these patients are treated by hemodialysis, peritoneal dialysis, and renal transplantation[3–6] Renal transplantation is the best choice for treatment of the ESRD,[6–14] and transplanted patients experience better survival rate and quality of life.[15,16] The first kidney transplantation in Iran was performed in 1967 in Shiraz.[4,17] The rate of renal transplantation in Iran has exceeded around 24 cases per every million persons in the recent years.[17] Deceased-donor transplantation is an important organ source,[18] but the survival rate of living donor transplantation is higher.[19–21] This study was designed to determine 10-year survival rate in patient with living donor transplantation in Shiraz transplant center, Namazi hospital, Shiraz, Iran, from January 1999 till December 2009.

## Materials and Methods

Our study is a survival rate analysis; subjects consist of all recipients of living donor kidney transplantation between January 1999 and December 2009. This center performed 1355 cases of kidney transplantation of which 843 were from living donors. As a rule, we do not use donors whose last preoperative serum creatinine level was over 1.4 mg/dl or last 24-hour urine output was less than 1 ml/kg/h. We have not been performing preoperative donor conventional or computed tomography (CT) angiography during the last 6 years of this period, and duplex ultrasonography and intravenous pyelography are the only imaging studies of the renal system of the donors that we use. CT angiography is used only when the duplex ultrasonography suggests any abnormal findings in renal vasculature. This selective use of contrast imaging studies prevents the use of excessive doses of intravenous contrast agents which may be toxic for the donor kidneys, and warrants the safety of the donor surgery and picks up an occasional donor with renovascular pathology. We prefer left kidney because of longer renal vein and better accessibility for nephrectomy, and use the right kidney only in special situations.

Intravenous methylprednisolone was used for induction immunosuppressive regimen for all patients, except for special patients such as those with previous graft failure due to rejection, second transplantation, or those with panel reactive antibody over 20% in which cases the methylprednisolone were replaced by basiliximab, daclizumab, or antithymocyte globulin, according to nephrologists’ preferences. Four different regimens had been prescribed to recipients for maintenance therapy:

1. Oral prednisolone, azathioprine (Imuran^®^), and cyclosporine (Neoral^®^).
2. Oral prednisolone, mycophenolate mofetil (Cellcept^®^), and cyclosporine (Neoral^®^).
3. Oral prednisolone, azathioprine which was changed to mycophenolate mofetil (Cellcept^®^) after different time intervals, and cyclosporine (Neoral^®^).
4. Oral prednisolone, mycophenolate mofetil (Cellcept^®^), and tacrolimus (Prograf^®^).

The exact time of transplantation was considered to be the ‘initial event,’ and irreversible loss of renal allograft (when the patient needs regular dialysis again) was defined as ‘end-point event.’ Cases, who did not encounter the end point event because of death from any other cause or those who were loss to follow-up, have been censored.

Data were collected through review of hospital and transplant clinic records. The organ survival and return to regular dialysis were assessed and determined by nephrologists and recorded in follow-up records of transplant clinics and related institutions such as ‘Management Center for Transplantation and Special Diseases’ and ‘Renal Patients Support Society.’

For analyzing survival rate, Kaplan-Meier method and for comparing survival curves Log-rank test were applied. Data modeling was done by applying Cox regression model and for evaluating assumption of hazard ratio proportionality (AHRP) (as one of the Cox model assumptions), two graphic models (i.e., plotting curve of Log (-log (t)) on log (t) and observed curve accompanied with predicted curve) and goodness of fit method were used.

Survival data were analyzed by running SPSS software, version 16 (SPSS Inc., Chicago, IL) and for evaluation of AHRP, Intercooled STATA 9 has been used.

## Results

As shown in Table 1, 61.4% of donors and 68.7% of recipients were males. About 52% of donors were nonrelated living donors and 39% were brothers or sisters of the recipient. Blood group O made up 52% and 42.4% of donors and recipients’ blood group, respectively. 82.9% of donors and recipients were of the same blood group.

**Table 1:** Distribution of demographic, surgical and medical variables in study

Variables	Subgroups	NO (%)	Variables	Subgroups	NO (%)
Donor’s gender	Male	518 (61.4)	Recipient’s gender	Male	579 (68.8)
	female	325 (38.6)		Female	263 (31.2)
Donor’s age	≤ 40 years	656 (77.8)	Recipient’s age	≤ 40 years	493 (58.5)
	> 40 years	187 (22.2)		> 40 years	350 (41.5)
Donor’s blood group	A	200 (23.8)	Recipient’s blood group	A	230 (27.3)
	B	183 (21.7)		B	218 (25.9)
	AB	21 (2.5)		AB	40 (4.7)
	O	438 (52)		O	355 (42.1)
Blood group	Same	698 (82.9)	Gender composition	Same	431 (51.2)
	Compatible	144 (17.1)		Compatible	411 (48.8)
HCV infection in recipients	Positive	7 (0.8)	Time to diuresis	Immediate	757 (94.7)
	Negative	836 (99.2)		Delayed	42 (5.3)
Cause of ESRD	Unknown	427 (52)	Creatinine level at	≤ 2 mg/dl	470 (90.4)
	Known	398 (48)	discharge	> 2 mg/dl	50 (9.6)
Donor source	Related	403 (47.8)	Anatomic position	Right	16 (2)
	unrelated	440 (52.2)		Left	796 (98)
Living related donor	Spouse	83 (20.5)	Immunosuppressive	1^st^ group	506 (61.5)
	Parents & baby	139 (34.5)	regimen	2^nd^ group	301(36.6)
	Sibling	157 (39)		3^rd^ group	10 (1.2)
	Other families	24 (6)		4^th^ group	6 (0.7)
Cold ischemiac time	≤ 2 hours	271 (91.2)	Cause of ESRD	Glomerulonephritis	103 (25.9)
	> 2 hours	26 (8.8)		Diabetes	74 (18.6)
				ADPKD*	31 (7.8)
				Obstructive	49 (12.3)
				Blood pressure	98 (24.6)
				Other causes	43 (10.8)

* ADPKD: Autosomal dominant polycystic kidney disease

In more than 50% of cases, underlying causes were not known. In 398 cases, the cause of disease was known, 103 cases (25.9%) of them were because of glomerulonephritis. Cold ischemia time was less than 1 hour in most of the cases. The mean age of the donors and recipients was 32.7 ± 8.6 and 35.2 ± 13.4 years, respectively. The duration of hospitalization after transplantation, duration of dialysis before transplant, and creatinine level at discharge were 12.2 ± 5.2 days, 14.2 ± 13.3 months, and 1.6 ± 1.1 mg/dl, respectively. 0.8% of recipients were HCV positive. 94.7% of recipients had urine production immediately after vascular declamping. In 98% of the procedures, the left kidney was used. Vascular complications were seen in 7.3% of all study patients. The most frequent vascular complications were hemorrhage seen in 5.2% of the cases followed by renal artery stenosis in 1.18%, renal artery thrombosis in 0.23%, and renal vein thrombosis in 0.35% of the patients. Of 843 patients, 5% were lost to follow-up. Of the remaining, 48 (5.7%) had irreversible transplant rejection. Mean duration of follow-up in this study was 53.07 ± 34.61 months.

**Figure 1:**
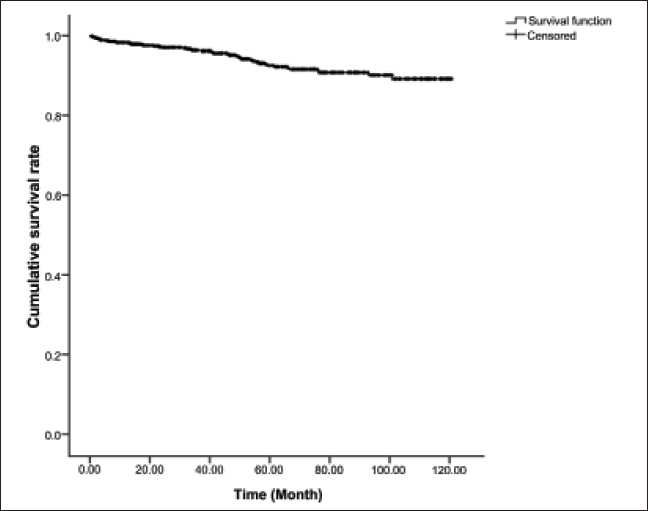
Allograft survival rate in renal transplant recipients from living donor

As shown in Figure 1, allograft survival rates at 1, 3, 5, 7, and 10 years after kidney transplantation were 98.3 ± 0.5, 96.4 ± 0.7, 92.5 ± 1.2, 90.8 ± 1.4, and 89.2 ± 1.8%, respectively.

Univariate analysis showed that graft survival rate in our series has not been significantly different in terms of the following factors: recipient’s place of residence, similarity of blood group, being HCV positive, sex of recipient, sexual mismatch of donors and recipients, age of recipient, type of immunosuppressive maintenance regimen, cause of ESRD, side of the donor graft, type of donor (related or unrelated), cold ischemia time, relationship of donor and recipient, time of first urination after vascular declamping, duration of dialysis before operation, and duration of hospitalization after surgery; whereas, blood group of donor (*P* = 0.02) and recipient (*P* = 0.015), sex of donor (*P* = 0.02), age of donor (*P* = 0.014), and creatinine level at discharge (*P* = 0.001) were significantly associated with graft survival.

**Figure 2:**
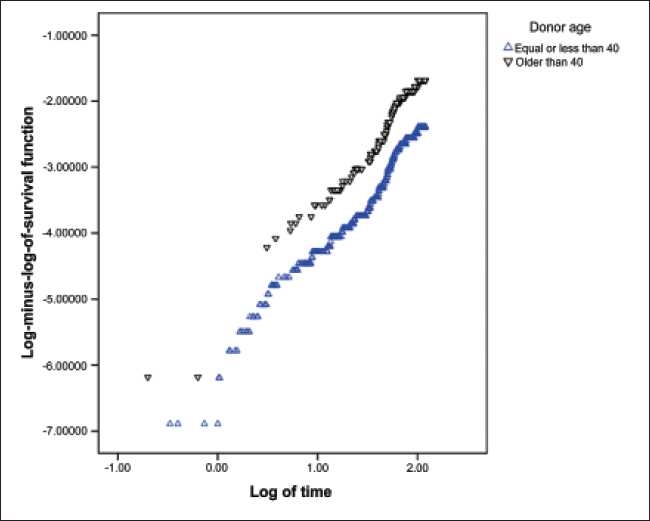
Testing of proportionality of hazard ratio assumption using the method of curve of Log (-log (t)) on log (t) based on donor’s age

**Figure 3:**
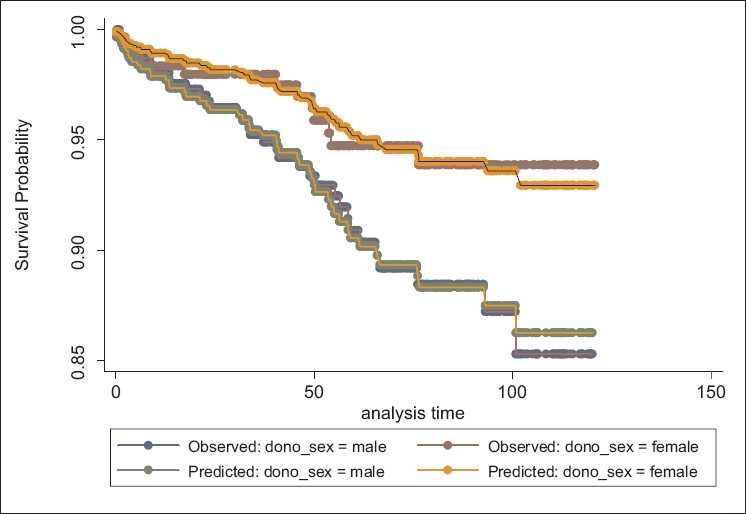
Assessment of proportionality of hazard ratio assumption using the method of predicted and observed values based on donor’s sex

Testing the AHRP using goodness of fit method showed that this assumption was true for variables. Graphic methods have been done for assessing this assumption for all variables, but because of space limitations of this article, these methods have been shown only for variables of donor’s age [Figure 2] and donor’s sex [Figure 3] as examples.

For modeling, those variables with P values less than 0.25 in univariate analysis and reasonable AHRP were entered into Cox proportional hazard model in a Forward stepwise manner. Results of Cox proportional hazard model have been shown in Table 2. The hazard ratio (HR) for female donors in comparison with males was 2.57 (*P* = 0.017, 95% confidence interval [CI] for HR = 1.18–5.4), and HR for over 40-year-old donors compared to those less than 40-year-old was 2.49 (*P* = 0.012, 95% CI = 1.22–5.06). Also, HR for creatinine level at discharge over 2 mg/dl was 4.9 (*P* = 0.001, 95% CI =2.17–10.5).

**Table 2:** Multivariate analysis by Cox Proportional Hazard Model

Variables	*P* value	Hazard ratio	95 %CI for Hazard ratio	
			Lower limit	Upper limit
Donor’s sex	0.017	1		
		2.57	1.18	5.4
Donor’s age > 40 years	0.012	1		
		2.49	1.22	5.06
Creatinine level at discharge >2 mg/dl	0.001	1		
		4.97	2.17	10.5

## Discussion

Thanks to progresses in surgical techniques and novel immunosuppressive drugs, the survival rate of transplantation has risen stunningly compared with past decades. According to the results of this study, 1-, 3-, 5-, 7-, and 10-year survival rates of renal transplantation in our center have been 98.3, 96.4, 92.5, 90.8, and 89.2%, respectively, whereas in this center and in the same period, allograft survival rates at 1, 3, 5, and 9 years after kidney transplantation from deceased donor was found to be 93.7, 89.1, 82.1, and 80.1%, respectively.[6]

Based on the previous reports of Iran organ procurement network,[6] nationwide one-year survival rate of renal transplantation is about 94.7% in Iran, and our study shows that this rate is slightly higher in our center.

Plenty of studies have shown that there is no significant relationship between sex of recipient and donor and survival rate of transplantation.[19,22,23] In our study, there was no significant relationship between sex of recipient and survival rate, but male donors there has statistically better graft survival rates.

According to our findings, there was no significant difference between graft survival from donors and recipients with same blood group with donors and recipients with compatible blood group. This finding is supported by some other studies,[24,25] but Park *et al*.[26] have showed that those with same blood group had better rates of survival in comparison to those with different blood groups.

Several studies[20,27] reported that rise of cold ischemia time leads to reduction of transplant survival rate significantly, but in a study done by Courtney *et al*.[19] there was no significant relationship between cold ischemia time and survival rate, a result which is similar with our study. One of the reasons for differences in results can emanate from defect in registering precise duration of cold ischemia time in our study, as just in 35% this time was known. In those with reported cold ischemia time, this time was less than 1 hour in most of the patients. These problems might affect the statistical power of the study.

Presence of HCV infection and its relationship with renal transplantation survival rate is still controversial. In some studies, graft and patient survival in HCV positives and negatives recepients have been reported to be equal.[28,29] On the other hand, some studies have evidenced higher rates of survival in HCV negative subjects.[20,30] From this point of view, our study also showed that there was no significant relationship between these two variables. But again, this conclusion may be due to low number (less than 1%) of HCV positive patients in our study group.

Courtney *et al*.[19] have shown that underlying cause of ESRD may affect renal transplantation survival rate. But some other studies did not report such a relationship.[22,24,25] There was no significant difference between transplantation survival rate and primary renal disease in our study, but it should be mentioned that in our study there was no known underlying reason for ESRD in half of our patients, because most of them referred for CKD evaluation with small nonfunctional kidneys without any known underlying disease, and diagnosing the cause in this group is almost impossible in most cases.

In our study, time of dialysis prior to renal transplantation had no significant relationship with survival rate, whereas some other studies[22,31] revealed that longer time on dialysis prior to transplantation is an independent predictor of worse graft survival rate.

In this study, the side of retrieved donor’s kidney has no significant effect on survival rate. As previously mentioned, in our center, we do not perform any angiographic evaluation of the donors and prefer to use left kidney of living donors because of having longer vein in comparison with right one. Because of low number of right kidneys, we could not rely on the statistical analysis of this factor.

Creatinine level at the time of discharge had a significant relationship with survival rate. In a study done by Rayhill *et al*., creatinine level at the time of discharge had significant relationship with renal transplant survival rate, with every 1mg/dl increase in creatinine level, HR of graft loss increases 1.8 unit.[32]

In recent decades, progresses in immunosuppressive regimes have led to increase in survival rate. First studies on drugs[33–36] indicated that mycophenolate, despite increasing expense, can reduce the acute graft rejection rate in comparison with azathioprine. But in recent studies, observed results have not shown any difference between these two drugs in terms of acute graft rejection, transplant survival rate, and patient survival.[37] Also, some studies have shown that there was no relationship between type of maintenance immunosuppressive drugs and survival rate[31,38] ; this finding is confirmed in our study.

Our study findings showed that donor age was affect graft survival, partly because of the reduction of nephron mass in older donors, but like other studies,[22,39] our study also did not show any significant relationship between age of recipient and survival rate.

Because of the lack of financial support we are not able to perform routine human leukocyte antigen typing in our recipients and also the acute rejection episodes after the first 3 months of operation are treated in nephrology wards (not transplant ward), and the data about these episodes are not complete. Therefore, we could not enumerate the effect of these variables on long-term survival of the graft.

## Conclusion

The 10-year graft survival rate of renal transplantation from living donor in this center is 89.2%, and graft survival rate in our cohort is satisfactory and similar to reports from large centers in the world. Findings of this study showed that graft survival was higher in donors younger than 40 years, male donors, and creatinine level less than 2 mg/dl at discharge.
